# Transcriptional Regulation Factors of the Human Mitochondrial Aspartate/Glutamate Carrier Gene, Isoform 2 (*SLC25A13*): USF1 as Basal Factor and FOXA2 as Activator in Liver Cells

**DOI:** 10.3390/ijms20081888

**Published:** 2019-04-16

**Authors:** Paolo Convertini, Simona Todisco, Francesco De Santis, Ilaria Pappalardo, Dominga Iacobazzi, Maria Antonietta Castiglione Morelli, Yvonne N. Fondufe-Mittendorf, Giuseppe Martelli, Ferdinando Palmieri, Vittoria Infantino

**Affiliations:** 1 Department of Science, University of Basilicata, 85100 Potenza, Italy; paolo.convertini@uky.edu (P.C.); simona.todisco@unibas.it (S.T.); ilaria.pappalardo8@gmail.com (I.P.); maria.castiglione@unibas.it (M.A.C.M.); giuseppe.martelli@unibas.it (G.M.); 2 Department of Biosciences, Biotechnologies and Biopharmaceutics, University of Bari, 70125 Bari, Italy; desantis.nutrizione@gmail.com (F.D.S.); ferdinando.palmieri@uniba.it (F.P.);; 3 Bristol Heart Institute, Bristol Medical School, University of Bristol, Bristol BS2 8HW, UK; domingaiacobazzi@live.it; 4 Department of Molecular and Cellular Biochemistry, University of Kentucky, Lexington, KY 40536, USA; y.fondufe-mittendorf@uky.edu

**Keywords:** SLC25A13, AGC2, gene expression, transcriptional regulation, FOXA2, USF1

## Abstract

Mitochondrial carriers catalyse the translocation of numerous metabolites across the inner mitochondrial membrane, playing a key role in different cell functions. For this reason, mitochondrial carrier gene expression needs tight regulation. The human *SLC25A13* gene, encoding for the mitochondrial aspartate/glutamate carrier isoform 2 (AGC2), catalyses the electrogenic exchange of aspartate for glutamate plus a proton, thus taking part in many metabolic processes including the malate-aspartate shuttle. By the luciferase (LUC) activity of promoter deletion constructs we identified the putative promoter region, comprising the proximal promoter (−442 bp/−19 bp), as well as an enhancer region (−968 bp/−768 bp). Furthermore, with different approaches, such as in silico promoter analysis, gene silencing and chromatin immunoprecipitation, we identified two transcription factors responsible for *SLC25A13* transcriptional regulation: FOXA2 and USF1. USF1 acts as a positive transcription factor which binds to the basal promoter thus ensuring *SLC25A13* gene expression in a wide range of tissues. The role of FOXA2 is different, working as an activator in hepatic cells. As a tumour suppressor, FOXA2 could be responsible for *SLC25A13* high expression levels in liver and its downregulation in hepatocellular carcinoma (HCC).

## 1. Introduction

The human aspartate/glutamate carrier isoform 2 (AGC2), encoded by the *SLC25A13* gene, is a member of the mitochondrial carrier family [1,2]. This family of transporters translocates a variety of metabolites across the mitochondrial membrane, thus linking biological and metabolic processes occurring in both mitochondria and cytosol [3,4]. Humans possess another isoform of the aspartate-glutamate carrier, AGC1, which is encoded by the *SLC25A12* gene. 

Both AGC1 and AGC2 exhibit a high substrate specificity for aspartate and glutamate and catalyse an electrogenic exchange of intramitochondrial aspartate^-^ for external glutamate plus a proton [5]. AGCs are essential for the malate/aspartate shuttle, which transfers the reducing equivalents of NADH plus H^+^ from cytosol to mitochondria [6] and is vital for aerobic glycolysis and alcohol metabolism, as well as for the supply of aspartate from the mitochondria to the cytosol, which is necessary for urea synthesis, purine and pyrimidine synthesis, protein synthesis and gluconeogenesis from lactate [7], especially in cells with negligible capacity for taking up aspartate from the blood, such as hepatocytes and neurons. Furthermore, the activity of AGCs in transfected mammalian cells is regulated by cytosolic Ca^2+^ reacting with AGCs on the external side of the mitochondrial membrane [8,9]. Thus AGCs, like the ATP-Mg/phosphate carriers [10,11,12,13], consist of two domains: the C-terminal domain containing all the sequence features of the mitochondrial carrier family members and the N-terminal domain protruding outside the mitochondrial matrix and containing EF (helix-loop-helix family)-hand Ca^2+^-binding motifs [5]. In spite of their common features, AGCs differ in their tissue-expression pattern—AGC1 is expressed at high levels only in heart, brain and skeletal muscle and AGC2 in many tissues and abundantly in the liver [14,15]. Furthermore, several studies highlighted distinctive functions of AGCs in normal and pathological conditions, which are largely but not completely explained by their different tissue expression. Mutations in *SLC25A12* are responsible for a disease characterized by developmental delay, epilepsy, hypotonia, hypomyelinization and decreased N-acetylaspartate in brain [16,17], whereas mutations in *SLC25A13* result in type II citrullinemia, which manifests with a dislike for carbohydrates, the inability to consume alcohol, citrullinemia and hyperammonemia leading to encephalopathy and neuropsyachiatric symptoms [18,19,20]. In particular, a specific involvement of AGC1 in hepatocellular carcinoma (HCC) and N-acetylaspartate synthesis in mice lacking AGC1 has been reported [21,22]. 

Due to the relevance of the mitochondrial carrier family members in metabolism, their activity must be regulated in different tissues and under various physiological and pathological conditions. It is not surprising, therefore, that the molecular mechanisms involved in gene expression regulation of several mitochondrial carriers, as well as the changes of their gene expression occurring during cell differentiation or in different tissues, diseases and cancer, have been thoroughly studied [23,24,25,26,27,28,29,30,31]. For example, it has been shown that in neuronal cells the gene expression of *SLC25A12* is primarily activated by the transcription factor CREB (cAMP response element binding protein), upregulated in neuronal differentiation and downregulated in neuroinflammation [32]. Furthermore, histone acetylation switches on *SLC25A12* gene in HCC cells [22]. In contrast, the molecular mechanisms regulating *SLC25A13* (AGC2) gene expression and its transcriptional control are still completely unknown.

In this work, we have investigated the transcriptional regulation of *SLC25A13* and identified two transcriptional factors, USF1 and FOXA2, that act as positive regulators of the *SLC25A13* gene expression. In particular, we have shown that FOXA2 works as a transcriptional activator and this might be a possible mechanism responsible for *SLC25A13* high expression levels in liver and downregulation in HCC as well as its expression pattern in different tissues.

## 2. Results

### 2.1. Screening of SLC25A13 Gene Promoter Activity

**Figure 1:**
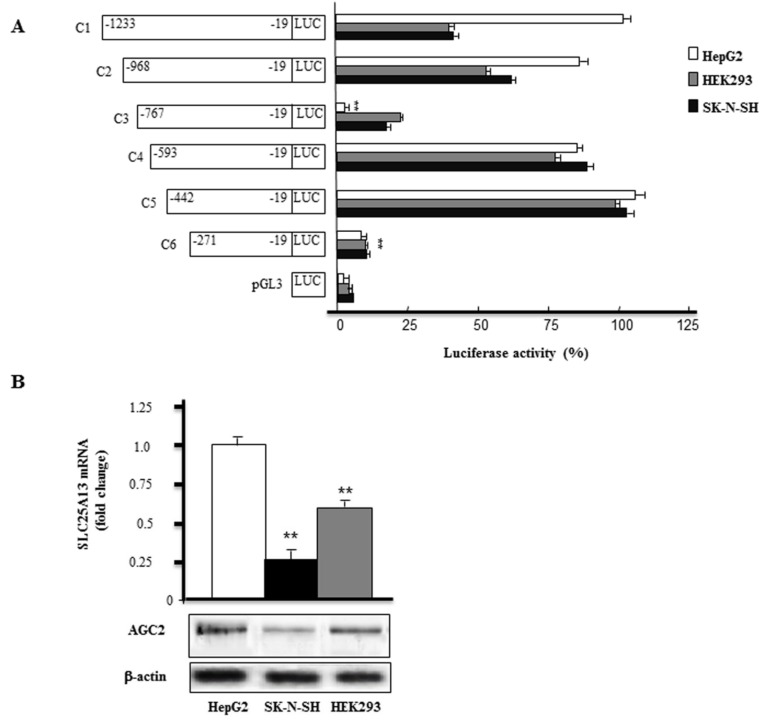
(**A**) Deletion analysis of the 5′-flanking region of the human *SLC25A13* gene. The deletion mutants named C1-C6 were cloned into the pGL3 basic-LUC vector and tested for LUC activity in transfected HepG2, HEK293 and SK-N-SH cells. pGL3 indicates the pGL3 basic-LUC vector alone. Numbering indicates the extent of fragments, while bars indicate LUC activity. The values of construct C1 transfected in HepG2 cells were set to 100%. Means ± SD of three duplicate independent experiments are shown. (**B**) Total RNAs extracted from HepG2, HEK293 and SK-N-SH cells were used to quantify SLC25A13 mRNA by real-time polymerase chain reaction (PCR). Means ± S.D. of four replicate independent real-time PCR experiments are shown. Where indicated, differences between samples and control were significant (**p < 0.01). AGC2 and β-actin proteins of HepG2, HEK293 and SK-N-SH cells were immunodecorated with specific antibodies. The Western blot analysis is representative of three independent experiments with similar results.

To analyse the promoter activity of the 5′-flanking region of the human SLC25A13 gene, various truncated versions of its promoter (from −1223 to −19 bp) were amplified and cloned into the luciferase (LUC) reporter vector pGL3 basic. These constructs were transfected into HepG2, SK-N-SH and HEK293 cell lines to determine the sequence elements required for SLC25A13 promoter activity. The results represented in Figure 1A show that the removal of the nucleotides from −968 to −768 bp (C3) displayed inhibited promoter activity though at varying levels. For instance, a reduction of luciferase activity of about 95% in HepG2 and about 25% in SK-N-SH and HEK293 cells, respectively, was found transfecting construct C3 compared to that of construct C1 transfected in HepG2 cells set to 100% (Figure 1A).

In all cell lines, deletion from −767 to −594 bp (C4) almost restored *SLC25A13* gene promoter activity that was further increased with the deletion of −593 to −443 bp (C5). Finally, deletion from −442 to −272 bp (C6) led to a drop in luciferase activity (Figure 1A) in HepG2 as well as in SK-N-SH and HEK293 cells. Because the luciferase activity driven by C5 is the highest within the deletion constructs and goes down significantly in all tested cell lines when driven by C6, we consider the region up to −272 bp as the proximal promoter containing regulatory elements able to drive the basal promoter activity of the human *SLC25A13* gene. In summary, we identified an activation domain in HepG2 cells (−968/−768 bp) and found that the −442/−272 bp fragment upstream transcription starting site contains a minimal promoter working in different cell lines.

Next, we performed an in silico analysis of the 1200 bp of the human SLC25A13 promoter region. This analysis revealed the lack of typical TATA box and the presence of a CCAAT box (−225/−229), the initiator element (INR) at −58/−51 bp, a downstream core promoter element (DPE) at −40/−35 bp, a canonical E-box (CACGTG) at –416/−411 bp as well as a number of putative cis-regulatory elements for several transcriptional factors. In addition, we measured SLC25A13 mRNA and AGC2 protein basal levels with real-time polymerase chain reaction (PCR) and Western-blot analysis, respectively, in HepG2, SK-N-SH and HEK293 cell lines. Figure 1B shows that HepG2 cells had the highest levels of AGC2 expression while SK-N-SH cells the lowest levels. Given that HepG2 cells showed the strongest promoter activity together with the highest endogenous expression of the SK-N-SH gene, we used these cells for further investigations about the molecular mechanisms involved in SLC25A13 gene expression regulation. 

### 2.2. Identification and Functionality of the USF1 Cis-Element in SLC25A13 Gene Promoter

**Figure 2:**
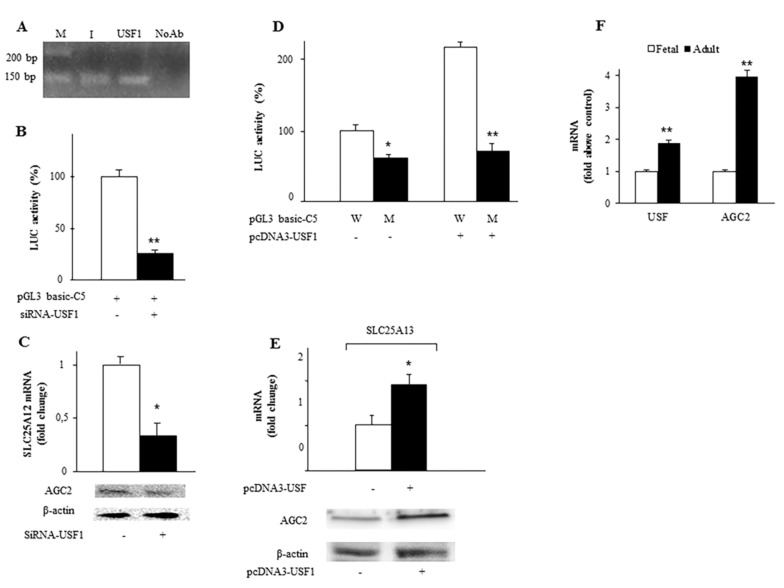
Effect of USF1 on *SLC25A13* gene expression. (**A**) ChIP-qPCR assay shows the amplification bands of PCR using the specific primers suitable to amplify the −498/−343 bp region of the *SLC25A13* gene promoter. M: Marker, lane I: Input, lane USF1: amplification PCR band after ChIP with specific antibody for human USF1, lane NoAb: without the anti-USF1 antibody addition. (**B**) Luciferase activity was measured in HepG2 cells co-transfected with C5 construct (−442/−19 of the *SLC25A13* gene promoter) and siRNA targeting human USF1 (+, black bar) or control siRNA (-, white bar). (**C**) Total RNA extracted from HepG2 cells treated without (white bar) or with (black bar) siRNA against human USF1 was used to quantify SLC25A13 mRNA by real-time PCR. Immunodecoration was performed with specific antibodies for AGC2 and β-actin proteins in HepG2 cells treated under the conditions described. (**D**) Luciferase activity of HepG2cells co-transfected with pGL3 basic-LUC vector containing the −442/−19 *SLC25A13* promoter region wild-type (W) or mutated (M) in USF1 site and pcDNA3-USF1 (+: black bar) or empty pcDNA3 (-: white bar). (**E**) Total RNA extracted from HepG2 cells transfected with pcDNA3-USF1 (+) or empty pcDNA3 (-) was used to quantify SLC25A13 mRNA by real-time PCR. AGC2 and β-actin of HepG2 cells transfected with pc-DNA3-USF1 (+) or empty pcDNA3 (-) were immunodecorated with anti-AGC2 and anti-β-actin antibodies. (**F**) Total RNA from liver foetal cells (white bar) and adult (black bar) was used to quantify USF1 and SLC25A13 mRNAs by real-time PCR. For panels (**B**–**F**), means ± SD of three duplicate independent experiments are shown; all differences between samples and relative controls were significant (*p < 0.05 or **p < 0.01 one-way ANOVA followed by Student’s *t*-test).

Given that the E-box motif usually interacts with the USF (Upstream Stimulating Factor) [33] in genes lacking TATA box [34], we checked for the contribution of E-box and USF in regulating *SLC25A13* gene expression. To this aim, different experiments were performed. First, we carried out a ChIP-qPCR (chromatin immunoprecipitation-PCR) analysis on chromatin extracted from HepG2 cells by using anti-USF1 antibody. As shown in Figure 2A, USF1 was bound to −498/−343 bp region of the SLC25A13 promoter. Without the anti-USF1 antibody addition, no immunoprecipitation was observed (Figure 2A, lane NoAb). Second, gene silencing experiments using siRNA targeting the human USF1 showed that luciferase activity, as well as AGC2 transcript and protein levels, were reduced as compared to those in the control cells (Figure 2B,C). Third, HepG2 cells were transfected with pGL3basic-C5 construct in the presence or absence of pcDNA3 vector containing the USF coding sequence. As shown in Figure 2D, luciferase activity was significantly enhanced in cells transfected with pGL3basic-C5 and pcDNA3-USF1 compared to cells transfected with pGL3basic-C5 alone. By contrast, no increase in luciferase activity was observed in cells transfected with the pGL3basic-C5 containing the mutated USF binding site (M), with or without the pcDNA3-USF1 expression vector (Figure 2D). Consistently, USF1 induced an increase of SLC25A13 mRNA and AGC2 protein (Figure 2E). Fourth, we performed gel-shift experiments by using wild-type and mutated E-box (−419/−409 bp) responsive element to support the presence—in this region of the *SLC25A13* gene promoter—of an active cis-element bound by HepG2 nuclear extracts (Supplementary Figure S1). These results clearly show that USF1 binds to the −419/−409 bp region of the human *SLC25A13* promoter probably working as an ubiquitous transcriptional factor.

Del Arco et al. showed that AGC2 was differently expressed during differentiation in rat liver [15]. Because USF1 is involved in transcription in development [33] we asked whether USF1 plays a role in AGC2 expression during liver development. For this, we measured the RNA levels of USF1 and AGC2 genes in foetal and adult liver cells (Figure 2F). Figure 2F shows a 1- and 3- fold overexpression of USF1 and AGC2 mRNAs, respectively, in adult compared to foetal liver tissue (set to 100%). These data suggest that USF1 could control *SLC25A13* gene expression during differentiation of liver cells.

### 2.3. Identification and Functionality of the FOXA2 Cis-Element in SLC25A13 Gene Promoter

**Figure 3:**
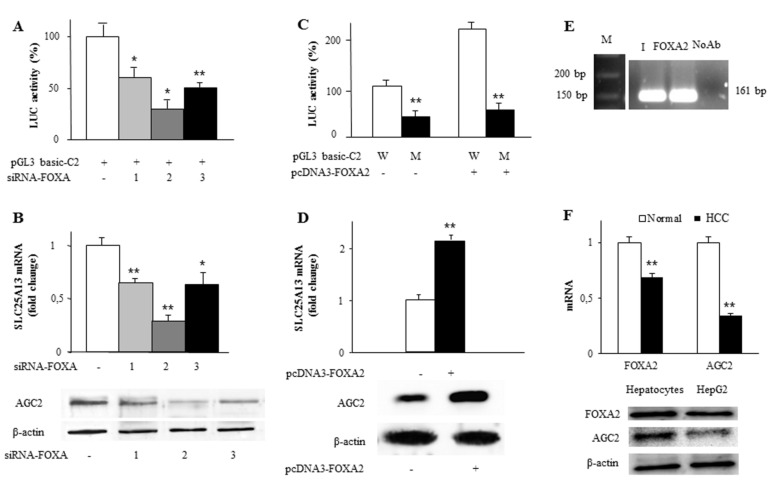
Effect of FOXA2 on *SLC25A13* gene expression. (**A**) HepG2 cells co-transfected with pGL3 basic-LUC vector containing the −968/−19 SLC25A13 gene promoter region and siRNA targeting human FOXA1, FOXA2 and FOXA3 or with control siRNA (-: white bar) were assayed for LUC activity. (**B**) Total RNA extracted from HepG2 cells transfected with siRNA targeting human FOXA1, FOXA2 and FOXA3 or with control siRNA (-: white bar) was used to quantify SLC25A13 mRNA by real-time PCR. AGC2 and β-actin proteins of transfected HepG2 cells were immunodecorated with specific antibodies. (**C**) HepG2 cells co-transfected with pGL3 basic-LUC vector containing the wild-type (W: white bar) or mutated (M: black bars) −968/−19 SLC25A13 gene promoter region wild- type and with pcDNA3-FOXA2 (+) or empty pcDNA3 (-) were assayed for LUC activity. (**D**) Total RNA extracted from HepG2 cells transfected with pcDNA3-FOXA2 (black bar) or empty pcDNA3 (white bar) was used to quantify SLC25A13 mRNA by real-time PCR. AGC2 and β-actin of transfected HepG2 cells were immunodecorated with specific antibodies. (**E**) ChIP-qPCR assay of FOXA2 binding to the endogenous SLC25A13 gene promoter shows the −1040/−864 bp region amplification band. M: Marker, lane I: Input, lane FOXA2: amplification PCR band after ChIP with a specific antibody for human FOXA2, lane NoAb: without the anti-FOXA2 antibody addition. (**F**) Total RNA extracted from normal (white bar) and hepatocellular carcinoma (HCC) (black bar) cells was used to quantify FOXA2 and SLC25A13 mRNAs by real-time PCR. FOXA2, AGC2 and β-actin proteins of primary hepatocytes and HepG2 cells were immunodecorated with specific antibodies. In (**A**–**D**) and (**F**) panels, means ± SD of three duplicate independent experiments are shown; all differences between samples and relative controls were significant (*p < 0.05 or **p < 0.01 one-way ANOVA followed by Student’s-*t*-test).

The construct C2, showing a high luciferase activity in HepG2 cells, contained a FOXA transcription factor element at −928/−918 bp. Since FOXA transcription factors are actively involved in the control of hepatic and pancreatic gene expression and metabolism [35], we focused our investigation on FOXA as a positive regulatory factor of *SLC25A13* gene expression. Firstly, HepG2 cells were transfected with siRNA targeting not only FOXA2 but also FOXA1 and FOXA3—the other members of FOXA family [36]—having tested that their silencing efficiency was of about 74%, 70% and 77%, respectively (Supplementary Figure S2). Seventy-two hours after transfection, the luciferase activity was reduced by about 40%, 70% and 50% in HepG2 cells transfected with siFOXA1, siFOXA2 and siFOXA3, respectively, as compared to control cells (Figure 3A). Consistently, when FOXA genes were silenced, both AGC2 transcript and protein levels were reduced in knockdown cells compared to wild-type HepG2 cells (Figure 3B). Given that FOXA2 gene silencing had the biggest impact on both *SLC25A13* gene promoter activity and ACG2 gene expression than the other FOXA genes, we focused our further analyses on this isoform. HepG2 cells were transfected with C2 construct in the presence or absence of pcDNA3 vector containing FOXA2 coding sequence (pcDNA3-FOXA2) and the relative luciferase activity was measured. Reporter activity was enhanced by about 100% in cells transfected with pGL3basic-C2 and pcDNA3-FOXA2 compared to cells transfected with pGL3basic-C2 alone (Figure 3C). In contrast, the luciferase activity was strongly reduced in HepG2 transfected with the mutated FOXA binding site (M) promoter-LUC vector, with or without the pcDNA3-FOXA2 expression vector (Figure 3C). Consistently, SLC25A13 mRNA and AGC2 protein significantly increased in HepG2 transfected with pcDNA3-FOXA2 (Figure 3D). Furthermore, electrophoretic mobility shift (EMSA) assay with wild-type and mutated FOXA responsive element further confirmed the specificity of DNA-binding to FOXA2 as a supershift was observed when a specific antibody against FOXA2 was used (Supplementary Figure S3). In addition, FOXA2 binding to the endogenous *SLC25A13* gene promoter was confirmed using the ChIP-qPCR assay. Figure 3E shows that chromatin extracted from HepG2 cells was immunoprecipitated by using a specific anti-FOXA2 antibody. These results clearly demonstrate that AGC2 expression is mainly affected by FOXA2 and the other isoforms exert a lower effect. Finally, we found a strong binding of FOXA2 transcription factor to −928/−918 bp region of the human *SLC25A13* required for *SLC25A13* gene activation (results not shown). All together these findings strengthen the link between SLC25A13 and FOXA2 expression patterns and suggest FOXA2 to be a transcriptional factor responsible for *SLC25A13* upregulation in liver.

Interestingly, Wang et al. [37] reported a low expression of FOXA2 in hepatocellular carcinoma. Since HepG2 are hepatocarcinoma cells we wondered whether the *SLC25A13* gene was also under-expressed in cancer. To this end, we measured expression levels of both FOXA2 and AGC2 in normal and hepatocellular carcinoma (HCC) cells. Figure 3F reveals a significant decrease in FOXA2 and AGC2 RNAs by about 30% and 65%, respectively, in cancer cells compared to normal cells. In HepG2 cells FOXA2 and AGC2 levels were also decreased as compared to normal liver cells.

### 2.4. Exogenous FOXA2 Induces Transcription of SLC25A13 in SK-N-SH Cells

**Figure 4:**
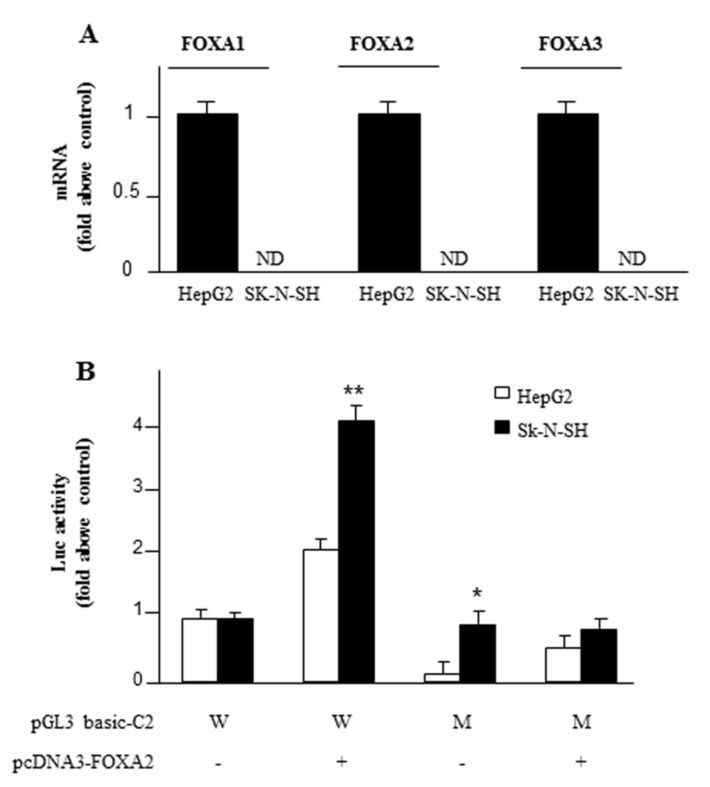
Activation of *SLC25A13* gene by FOXA2. (**A**) FOXA1, FOXA2 and FOXA3 mRNA levels in HepG2 and SK-N-SH cells. (**B**) HepG2 and SK-N-SH cells co-transfected with pGL3 basic-LUC vector containing C2 construct (968/−19 of the *SLC25A13* gene promoter region) wild-type (W) or mutated (M) in FOXA2 binding site and with pcDNA3-FOXA2 (+: black bars) or empty pcDNA3 (-: white bar) were assayed for LUC activity. Where indicated, differences between samples and relative controls were significant (*p < 0.05 or **p < 0.01 one-way ANOVA followed by Student’s *t*-test).

To further confirm the specificity of FOXA2 as an activator of the *SLC25A13* gene we overexpressed FOXA2 in SK-N-SH. First, we checked that none of the FOXA family isoforms (FOXA1, FOXA2 and FOXA3) were present in SK-N-SH. Indeed, real-time PCR performed on mRNA extracted from SK-N-SH cells showed the absence of FOXA1, FOXA2 and FOXA3 transcription factors (Figure 4A). In these experiments, HepG2 cells were taken as the reference for each isoform. Second, upon expression of FOXA2 in SK-N-SH, the luciferase activity—relative to the SLC25A13 gene promoter—increased by four times compared to the cells transfected with pGL3-basic alone (Figure 4B). This effect was abolished using the construct harbouring a mutated FOXA2 responsive element (Figure 4B). These findings clearly indicate that FOXA2 works as a strong activator of *SLC25A13* gene expression in various cell lines.

### 2.5. Synergy between FOXA2 and USF1 in Regulating SLC25A13 Gene Expression and AGC2 Function 

**Figure 5:**
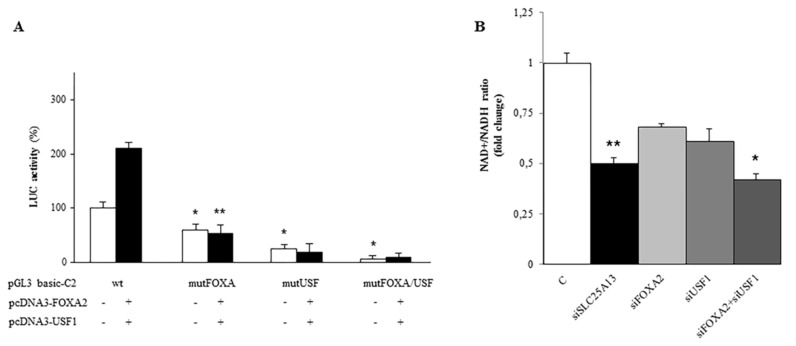
(**A**) Synergy between FOXA2 and USF1. HepG2 cells co-transfected with pGL3 basic-LUC vector containing C2 construct (−968/−19 of the *SLC25A13* gene promoter region) wild-type (wt) or mutated (mutFOXA, mut USF and mut FOXA/USF) in combination with pcDNA3-FOXA2 and pcDNA3-USF1 (+: black bars) or empty pcDNA3 (-: white bars) were assayed for LUC activity. (**B**) FOXA2 and USF1 effect on AGC2 activity. Quantification of the NAD^+^/NADH ratio in HepG2 cells transfected with siRNA against FOXA1 and USF1 alone or in combination. siRNA against *SLC25A13* was used as control. Where indicated, differences between samples and relative controls were significant (*p < 0.05 or **p < 0.01 one-way ANOVA followed by Student’s *t*-test).

The results presented above indicate a primary role for FOXA2 and USF1 transcription factors in the regulation of *SLC25A13* gene expression. We therefore wondered if FOXA2 and USF1 interplay in activating *SLC25A13* transcription and in turn regulating AGC2 function. To this end, we transfected HepG2 cells with C2 construct—containing binding sites for both FOXA2 and USF1 factors—wild-type or mutated in FOXA or UFS sites alone or together and measured LUC activity under each condition. *SLC25A13* promoter activity strongly decreased when a single site was mutated and was almost absent in the presence of a double mutation (Figure 5A, white bars). When C2 construct was transfected together with pcDNA3-FOXA2 and pcDNA3-USF1, we observed a great increase of LUC activity by about 100% (Figure 5A wt, black bars). This effect was abolished in the presence of a single mutation at level of FOXA or USF binding site (Figure 5A mutFOXA or mutUSF black bars). Thus, a single mutation in one of these binding sites is enough to completely abolish SLC25A13 promoter activity. These results support a collaborative interplay of both FOXA2 and USF1 in driving high levels of SLC25A13 gene expression.

Next, we investigated the effect of FOXA2 and USF1 on AGC2 activity. As AGC2 is an essential component of the malate/aspartate shuttle which transfers the reducing equivalents of NADH plus H+ from cytosol to mitochondria [6], we measured the NAD^+^/NADH ratio in HepG2 cells transfected with siRNA against FOXA1 and USF1 alone or in combination. As a control, we silenced *SLC25A13* and observed a significant decrease in NAD^+^/NADH ratio by about 50% in agreement with the role of AGC2 in regulating NADH redox status (Figure 5B). When FOXA2 or USF1 genes were silenced we also obtained a great reduction in NAD^+^/NADH ratio; this reduction was even greater in the presence of FOXA2/USF1 double gene silencing (Figure 5B). These findings suggest a role for FOXA2 and USF1 as regulators of AGC2 activity thus implying their control of the NADH redox status.

## 3. Discussion

The physiological role of AGC2 and its different tissue distribution (particularly high in liver) suggest that its expression might be regulated at a transcriptional level in a specific tissue and cell-type manner. In order to elucidate human *SLC25A13* gene regulation we focused our investigation on the 1233 bp upstream of the ATG codon, where we identified the *SLC25A13* promoter region. The basal promoter activity of *SLC25A13* is contained in a 442 bp fragment upstream of the transcriptional start site, as confirmed by the reduction of luciferase activity when this region was deleted from the experimental constructs employed in HepG2, SK-N-SH and HEK293 cell lines. The *SLC25A13* promoter structure is interesting as both INR and E-box (CACGTG) sequences interact with HLH transcription factors, such as USFs, in TATA-less and pyrimidine-rich promoter [38,39]. Consistent with this promoter model structure, our findings show a functional binding of USF1 to the E-box both in-vivo and in EMSA experiments. Interestingly, the USF binding site is present in both human and rat mitochondrial aspartate/glutamate carrier isoform 2 gene promoters. The USFs bHLH-leucine zipper transcription factors are ubiquitously expressed and act as key regulatory factors of the transcriptional machinery mediating recruitment of chromatin remodelling enzymes, interacting with co-activators and members of the pre-initiation complex [40]. Given that an E-box motif binding USF1 is located in the region corresponding to the basal promoter activity, it is likely that the USF1 factor is involved in the initial steps of *SLC25A13* gene transcription, such as the pre-initiation complex. However, USF1 could also be involved in coordinating the regulation of lipid and glucose metabolism by a mechanism not yet understood. USF1 is decreased by fasting and increased by refeeding [41]. In culture cells, high extracellular glucose levels enhance USF1 expression [42,43,44,45]. Increase of USF expression in glucose treated cells is consistent with the role of AGC2 in glycolysis because, as component of the malate/aspartate shuttle, AGC2 transfers the reducing equivalents of NADH plus H^+^ from cytosol to mitochondria [6]. Furthermore, our data also show differential expression of *SLC25A13* in foetal (low) and adult (high) cells in parallel with differential expression USF. It might be speculated that increasing expression of USF in adult cells compared to foetal cells also affects AGC2 expression.

It is known that USFs work in tandem with general or specific transcription factors, including FOXAs [46,47]. By means of different approaches including ChIP, gene silencing and in silico analysis we identified the promoter sequence located at −928 bp to −918 bp as the binding site for FOXA2. Indeed, also other FOXA family members (FOXA1 and FOXA3) affect the *SLC25A13* gene expression, although to lower extent than FOXA2. The FOXA subfamily of winged helix/forkhead box (Fox) transcription factors is involved in the regulation and differentiation of metabolic tissues, such as liver, pancreas and adipose tissue, acting as factors, the binding to promoters of which enables chromatin access to other tissue-specific transcription factors [48]. In particular, FOXA2 is essential for glucose and lipid homeostasis [49,50]. In fact, several genes encoding hepatic enzymes involved in metabolism during fasting and energy deprivation contain FOXA2-binding sites [51,52,53]. Examples include the gluconeogenic enzymes phosphoenopyruvate carboxychinase (PepCk) [54], glucose-6-phosphatase (G6pc) [55] and tyrosine aminotransferase (Tat) [56], as well as enzymes of lipid catabolism, such as carnitine palmitoyltransferase 1, carnitine acylcarnitine translocase, hydroxyacyl-CoA dehydrogenase and lipoprotein lipase and of ketogenesis, such as 3-hydroxy-3-methylglutaryl-CoA synthase 1 [24,50]. Our results further suggest that FOXA2 does not contribute to *SLC25A13* gene expression in some non-hepatic cells, such as SK-N-SH cells. This contention is supported by the experimental observations reported in this study and in particular by the lack of FOXA transcription factors in SK-N-SH cells and by the very high increase in gene reporter activity obtained by FOXA2 overexpression in these cells. Therefore, this study may explain at the molecular level, at least in part, the differences in AGC2 expression levels between liver and other tissues [57]. Interestingly, we also detected the presence of FOXA2 binding site in the rat (Rattus Norvegicus) slc25a13 gene promoter and its absence in the AGC1/SLC25A12 gene promoter. Furthermore, identification of FOXA2 as specific enhancing protein might be useful as therapeutic target to increase the expression of AGC2 in patients with type II citrullinemia showing a mild phenotype, as reported for other mitochondrial carrier deficiencies [58]. 

In the light of our findings, it is clear that USF1 and FOXA2 are positively regulators of *SLC25A13* gene expression. It is likely that both factors interact each other as cotransfection of a FOXA2 binding site mutated version with both USF1 and FOXA2 transcription factors causes no increase of SLC25A13 promoter activity. Noteworthy, USF1 and FOXA2 single or double gene silencing significantly reduces the NAD^+^/NADH ratio, that is, negatively affects AGC2 function by regulating its transcription. Obviously, it cannot be excluded that other co-factors and transcription factor-binding sites participate in the regulation of the *SLC25A13* gene expression, beyond the central role played by FOXA2 and USF1.

This study sheds some light on the mechanisms underlying the *SLC25A13* gene expression which result to be very different than those controlling *SLC25A12* at least in hepatocarcinoma. AGC2 (at the level of mRNA and protein) is significantly downregulated in liver cancer cells, whereas AGC1 is upregulated by an epigenetic mechanism consisting in histone hyperacetylation [22]. Our findings demonstrate that FOXA2 downregulates *SLC25A13* expression and function, in agreement with the reported role of FOXA2 as tumour suppressor in HCC [59,60]. In this regard, Oncomine (https://www.oncomine.org) database used for an in-depth analysis of SLC25A13 and FOXA2 mRNA levels reveal a strong downregulation of both genes in different types of tumours (Affimetrix Human Genome HT U133A Array, Human Genome U133A 2.0 Array) when compared to normal tissues [61,62,63]. So, it can be hypothesized that FOXA2 gene expression decreases in HCC and in turn reduces SLC25A13 transcription levels. 

The reasons for *SLCA25A13* downregulation in HCC are not clear. However, it appears that AGC2 is not essential for cancer cells’ proliferation as *SLCA25A13* gene silencing does not affect HCC cell proliferation [22]. On the contrary, SLCA25A12 gene silencing significantly affects liver cancer cell growth and migration suggesting a specific role for AGC1 in HCC [22]. Thus, it is likely that both AGC1 and AGC2 are implicated in the malate/aspartate shuttle but AGC1 plays also a more specific role in the biosynthesis of nucleotides [22]. As cancer cells rely on lactate dehydrogenase and cytosolic malate dehydrogenase 1 in regenerating cytosolic NAD+ [64], a downregulation of *SLC25A13* does not compromise HCC cell survival. Moreover, preliminary quantification data by NMR in *SLC25A13* silenced HCC cells indicate a decrease of glutamate levels compared to control cells (data not shown), suggesting that *SLC25A13* gene silencing induces the conversion of glutamate in α-ketoglutarate via mitochondrial glutamate dehydrogenase (GDH) thus sustaining TCA cycle.

In conclusion, we have characterized the promoter of the human *SLC25A13* gene and have demonstrated, for the first time, that the transcriptional factors FOXA2 and USF1 are involved in the positive regulation of *SLC25A13* gene transcription. Furthermore, we have shown that FOXA2 works as an enhancing factor and this is most likely the mechanism responsible for *SLC25A13* high expression levels in liver and its downregulation in HCC.

## 4. Materials and Methods 

### 4.1. Construction of Plasmids.

Progressive deletion fragments of the −1233/−19 bp region of the AGC2 gene promoter, with or without mutations in FOXA or USF binding sites, were obtained by PCR. They were cloned into the pGL3 basic-LUC vector upstream of the LUC gene coding sequence (Promega Madison, WI, USA). FOXA2 (pcDNA3-FOXA2) and USF1 (pcDNAs-USF1) expression vectors were obtained by cloning the human FOXA2 cDNA (Accession No. NM_004496.2) and the human USF1 cDNA (Accession number NM_007122), respectively, into the pcDNA3.1 vector (Thermo Fisher Scientific, San Jose, CA, USA). 

The sequences of primers used to generate the mutated FOXA and USF sequences of the SLC25A13 gene in the −932/−909 bp and −424/−404 bp regions, respectively, are shown in Table 1.

### 4.2. Cell Culture, RNA Interference and Transient Transfection.

HepG2 and HEK293 cells (Sigma–Aldrich, St Louis, MO, USA) were maintained in high glucose DMEM (Dulbecco’s modified Eagle’s medium) containing 10% (*v*/*v*) fetal bovine serum, 2 mM L-glutamine, 100 U penicillin and 100 lg/mL streptomycin at 37 °C in 5% CO_2_. SK-NSH cells (ICLC, Interlab Cell Line Collection) were grown in RPMI 1640 medium (Roswell Park Memorial Institute) at 37 °C in 5% CO_2_. Primary human hepatocytes were grown in a hepatocyte culture medium according to the manufacturer’s instructions (Lonza, Walkersville, MD, USA). Transient transfection was performed as reported [65] using 0.5 μg of pGL3 basic-LUC vector containing the full-length −1233/−19 bp region of the AGC2 gene promoter or deletion fragments of this region and 10 ng of pRL-CMV (Promega) to normalize the extent of transfection. Transfected cells were assayed for LUC activity using the Dual-Luciferase^®^ Reporter Assay System (Promega). The luminescence was measured using GloMax plate reader (Promega).

For USF1 and FOXA2 overexpression, HepG2 cells were transfected using 0.5 μg of pcDNA3–USF1 or pcDNA3-FOXA2 vector or both. In RNA interference experiments, the specific pre-designed small interfering RNAs (siRNAs) targeting human FOXA1 (s6689, Thermo Fisher Scientific), FOXA2 (s6691, Thermo Fisher Scientific) and FOXA3 (s6693, Thermo Fisher Scientific) were transfected in HepG2 cells using siPORT NeoFX Transfection Agent (Thermo Fisher Scientific). A siRNA (Cat. No. C6A-0126, Thermo Fisher Scientific) with no significant similarity to human, mouse or rat gene sequences was used as negative control. 

### 4.3. Reverse Transcriptase-PCR and Real-Time PCR

Total RNA was extracted from 1 × 10^6^ HepG2, HEK293 and SK-N-SH cells or purchased HCC cells (Rockville, MD, USA); normal adult and foetal liver RNAs were purchased from Origene (Rockville, MD, USA); and reverse transcription was performed as reported [66]. Real-time PCR was conducted as previously described [67]. Assay-on-demand for human AGC2 (Hs00185185_m1), human FOXA1 (Hs00293689_s1), human FOXA2 (Hs00232764_m1), human FOXA3 (Hs00270130_m1) and human β-actin (4326315E) were purchased from Thermo Fisher Scientific. All transcript levels were normalized against the β-actin expression levels. To this end, the difference between β-actin Ct value and the target gene Ct value was used to generate a ΔCt value. ΔΔCt was calculated by subtracting the mean value of ΔCt of the control from ΔCt value. Finally, fold changes in each gene expression were calculated via the comparative 2−ΔΔCt method using the formula: 2−ΔΔCt = ΔCtBS patient − ΔCtcontrol.

### 4.4. Western Blotting

Western blot analysis was performed as reported previously [68]. Briefly, proteins were electroblotted onto nitrocellulose membranes (Bio-Rad, Hercules, CA, USA) and treated with anti-AGC2 (sc-100937, Santa Cruz Biotechnology, Santa Cruz, CA) or anti-β-actin (sc-47778, Santa Cruz Biotechnology) antibody. The immunoreactions were detected by the Immobilon western ECL system (Millipore, Burlington, MA, USA). Immunolabeled protein bands were analysed by densitometry using ImageJ quantitative software (NIH, Bethesda, MD, USA) and normalized to β-actin levels.

### 4.5. Chromatin Immunoprecipitation

ChIP experiments were performed as previously reported [69]. Briefly, 2 × 10^7^ of HepG2 cells were fixed by 1% formaldehyde at 37 °C for 10 min; afterwards, the cells were lysed and sheared by sonication in a 1% SDS lysis buffer to generate cellular chromatin fragments of 400–500 bp. The chromatin was immunoprecipitated for 14–16 h at 4 °C using specific antibodies to FOXA2 (sc-6554X, Santa Cruz Biotechnology) and to USF1 (sc-229X Santa Cruz Biotechnology). After reverse cross-linking, chromatin immunoprecipitates were purified, then 2 µL of each sample was analysed by PCR (35 cycles) using a forward FOXA2 primer (5′-CCTGATCACCACTGTCTTAT-3′) and a reverse primer (5′-CCGGGTCAAAAGACTTCC-3′) suitable to amplify the −1040/−864 bp region of the SLC25A13 promoter and a forward USF1 primer (5′-CCTCAGGTGACGGGCTTG-3′) and a reverse primer (5′-GTGATCCAGCGGCCTCTG-3′) suitable to amplify the −498/−343 bp region of the SLC25A13 promoter.

### 4.6. Intracellular NAD+/NADH Quantification

NAD+/NADH ratio was determined by using a NAD/NAD Quantification Kit (Sigma–Aldrich) according to the manufacturer’s instructions. Briefly, HepG2 cells were seeded in 24-well plates and gene silencing experiments were carried out as described above. Wells were washed once with ice cold PBS. For each assay a pellet from 2 x10^5^ cells obtained by centrifuging at 2000 rpm for 5 min was used. Afterwards, cells were lysed in NADH/NAD Extraction Buffer by freeze/thawing for 2 cycles of 20 min on dry ice followed by 10 min at room temperature. Then samples were centrifuged at 13,000 rpm for 10 min to remove insoluble material. Part of the cell lysate was incubated at 60 °C for 30 min to produces NADH. Enzyme mix cycling buffer and developer were added according to the manufacturer’s protocol. Plates were incubated for 3 h and absorbance was read at 450 nm using the GloMax plate reader (Promega).

### 4.7. Statistical Analysis

Results are presented as means ± S.D. of, at least, four independent experiments. Differences between means of samples were compared using the one-way ANOVA test. Subsequently, where indicated, the Student’s t-test was used as a post hoc test to compare differences between means of samples and relative controls. A 5% or 1% significance level and 95% (*) or 99% (**), respectively, confidence intervals were used in the statistical analysis.

## Figures and Tables

**Table 1:** List of primers used to generate the mutated FOXA and USF binding sequences.

Name	Sequence
wtFOXA	5′-TGCTTGTTTATTTATTTTAGTAGG-3′
mutFOXA	5′-TGCTTGGGGAGGGATGGTAGTAGG-3′
wtUSF	5′-GCGGGGTCACGTGTCCCTGT-3′
mutUSF	5′-GCGGGGTACCTGTTCCCTGT-3′

* Mutated nucleotides are underlined.

## Supplementary Materials

Supplementary materials can be found at https://www.mdpi.com/1422-0067/20/8/1888/s1.
