# Supplementary material for: Transcriptional Regulation Factors of the Human Mitochondrial Aspartate/Glutamate Carrier Gene, Isoform 2 (SLC25A13): USF1 as Basal Factor and FOXA2 as Activator in Liver Cells
Source: Int J Mol Sci. 2019 Apr 16;20(8):1888. doi: 10.3390/ijms20081888 (PMC6515469; doi:10.3390/ijms20081888)
Supplement: Supplementary file 1 [file ijms-20-01888-s001.pdf]

Supplementary Figures

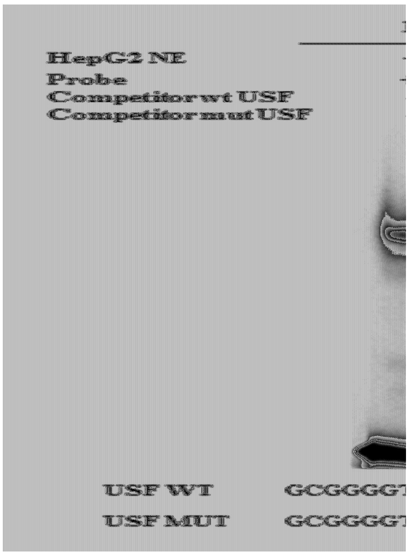

Supplementary Figure S1: USF1 binding in EMSA experiment. USF1 binding to the radiolabeled probe corresponding to -424/-404 bp of the SLC25A13 gene promoter region. Five µg of HepG2 cell nuclear extract proteins were incubated with the radiolabeled probe and in the presence and absence of unlabeled specific (WT) or mutated (MUT) competitor in 100-fold molar excess. The sequences of WT and MUT competitor are indicated in figure.

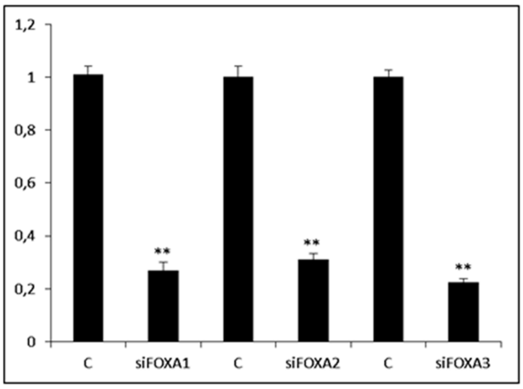

Supplementary Figure S2: FOXA1, FOXA2 and FOXA3 gene silencing efficiency. HepG2 cells were transfected with siRNA targeting FOXA1 (siFOXA1), FOXA2 (siFOXA2), FOXA3 (siFOXA3), or control scramble siRNA (C) and used to quantify FOXA1, FOXA2 and FOXA3 mRNAs. Means ± S.D. of four replicate independent real-time PCR experiments are shown. All differences between samples and relative controls were significant (**$p < 0.01$ one-way ANOVA followed by Student's-t-test).

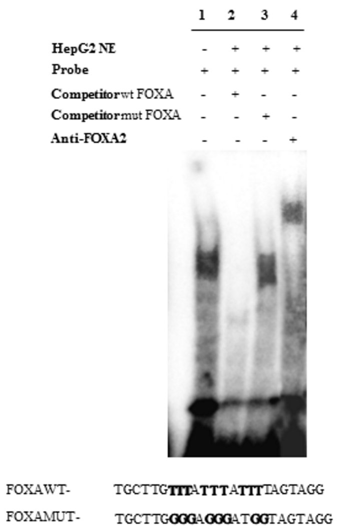

Supplementary Figure S3: FOXA2 binding in EMSA supershift experiment. FOXA2 binding to the radiolabeled probe corresponding -932/-909 bp of the SLC25A13 gene promoter region. Five µg of HepG2 cells nuclear extract proteins were incubated with the radiolabeled probe and in the presence and absence of unlabeled specific (WT) or mutated (MUT) competitor in 100-fold molar excess. The sequences of WT and MUT competitor are indicated in figure. Lane 4 shows the supershift in the presence of specific antibody against FOXA2.
